# Patterns of Change in Collaboration Are Associated with Baseline Characteristics and Predict Outcome and Dropout Rates in Treatment of Multi-Problem Families. A Validation Study

**DOI:** 10.3389/fpsyg.2017.01221

**Published:** 2017-07-21

**Authors:** Egon Bachler, Alexander Fruehmann, Herbert Bachler, Benjamin Aas, Marius Nickel, Guenter K. Schiepek

**Affiliations:** ^1^PMU Institute Synergetic and Psychotherapy Research Salzburg, Austria; ^2^Institute for Psychoanalysis and Familytherapy Salzburg, Austria; ^3^General Medicine and Family Medicine, Medical University of Innsbruck Innsbruck, Austria; ^4^Faculty of Psychology and Educational Sciences, Ludwig-Maximilians-Universität München Munich, Germany; ^5^Clinic for Psychiatry, Medical University of Graz Graz, Austria

**Keywords:** collaboration, SES, home-based treatment, therapy outcome, outcome measures

## Abstract

**Objective:** The present study validates the Multi-Problem Family (MPF)-Collaboration Scale), which measures the progress of goal directed collaboration of patients in the treatment of families with MPF and its relation to drop-out rates and treatment outcome.

**Method:** Naturalistic study of symptom and competence-related changes in children of ages 4–18 and their caregivers.

**Setting:** Integrative, structural outreach family therapy.

**Measures:** The data of five different groups of goal directed collaboration (deteriorating collaboration, stable low collaboration, stable medium collaboration, stable high collaboration, improving collaboration) were analyzed in their relation to treatment expectation, individual therapeutic goals (ITG), family adversity index, severity of problems and global assessment of a caregiver’s functioning, child, and relational aspects.

**Results:** From *N* = 810 families, 20% displayed stable high collaboration (*n* = 162) and 21% had a pattern of improving collaboration. The families with stable high or improving collaboration rates achieved significantly more progress throughout therapy in terms of treatment outcome expectancy (*d* = 0.96; *r* = 0.43), reaching ITG (*d* = 1.17; *r* = 0.50), family adversities (*d* = 0.55; *r* = 0.26), and severity of psychiatric symptoms (*d* = 0.31; *r* = 0.15). Furthermore, families with stable high or improving collaboration maintained longer treatments and had a bigger chance of finishing the therapy as planned. The odds of having a stable low or deteriorating collaboration throughout treatment were significantly higher for subjects who started treatment with low treatment expectation or high family-related adversities.

**Conclusion:** The positive outcomes of homebased interventions for multi-problem families are closely related to “stable high” and an “improving” collaboration as measured with the MPF-Collaboration Scale. Patients who fall into these groups have a high treatment outcome expectancy and reduce psychological stress. For therapeutic interventions with multi-problem families it seems beneficial to maintain a stable high collaboration or help the collaboration, e.g., by fostering treatment expectation.

## Introduction

The principles of therapeutic change can be divided into three groups: client factors, relational aspects and components concerning therapeutic techniques (Castonguay and Beutler, 2006, p.8). Client factors represent prognostic factors such as attachment style, gender or type and severity of pathology. Additional moderating variables of patients contain resistance, functional impairment, stages of change, expectations, etc. The second group – relational aspects – includes, e.g., therapeutic alliance, empathy, goal consensus and goal-directed collaboration, feedback, repair of alliance ruptures or management of countertransference. The third group of technical factors adds such factors as the level of therapist directedness, treatment intensity (length, frequency, multi-modal, etc.). The therapeutic change that is accomplished by the interaction of these three therapeutically relevant groups has been shown to often run discontinuously through different “stages of change” (preparation, action, maintenance, contemplation, action) (Emmerling and Whelton, 2009; Schiepek et al., 2015). The crucial step is from “contemplation to action,” i.e., to “collaboration.”

Recently, research has taken interest in the second group of relational aspects of therapy, especially therapeutic relationship. An exact definition of therapeutic relationship and its underlying components is, however, not easily given. Some research dissects therapeutic alliance, goal consensus, collaboration, etc., into different aspects (Castonguay and Beutler, 2006; Anderson and Johnson, 2010; Norcross and Wampold, 2011). Others combine such factors conceptually, beginning with early research which understood therapeutic alliance as the combination of three factors: affective bond between client and therapist and mutual agreement or collaboration on goals and methods (Bordin, 1994; Stackert and Bursik, 2006; Tryon and Winograd, 2011; Asnaani and Hofmann, 2012; Accurso et al., 2013). Recent research confirms these factors (Johansson and Jansson, 2010; Munder et al., 2010) and finds consistent associations between the therapeutic relationship, individual goals and treatment outcome for various types and contexts of child and adolescent therapy (Shirk and Karver, 2003; Jacob et al., 2017a).

Improvements with respect to patient expectancies on outcome foster collaborative aspects of the working alliance (Falkenstrom et al., 2013) and reciprocally influence each other (Xu and Tracey, 2015). Johansson et al. (2011) described “expectancy–alliance–outcome” as a general mediational chain. Together with other common factors, a non-linear and individual interdependent mediating line in effective therapeutic processes emerges. Swift and Derthick (2013) demonstrated the relationship between increasing hope and treatment outcome. They showed specific interventions to contribute to patient outcome expectation by presenting a convincing treatment rationale, increasing clients’ faith in their therapists, expressing faith in clients, providing outcome education, and comparing progress with expectations.

The difficulty of discriminating the different concepts and establishing a hierarchy in the realm of therapeutic relationship becomes especially apparent when creating or using instruments for measurement. Many items meant to distinctively measure therapeutic relationship tend to load on many factors (Horvath et al., 2011) and separate measures of, e.g., therapeutic alliance (Horvath and Greenberg, 1989; WAI Working Alliance Inventory) and collaboration correlate highly (Hatcher and Barends, 1996; CASF, The confident Collaboration scale of the patient). In a meta-analytical study, it has been shown that keeping mutually developed individual goals in focus is a good predictor of outcome, with an effect size for “goal directed collaboration” of *d* = 0.69; *r* = 0.33 (Tryon and Winograd, 2011). Different patterns of collaboration (improving, stable high, and deteriorating collaboration) have been shown. These groups generally manifest differences with respect to outcome rates in different treatment settings (Polaschek and Ross, 2010).

While the empirical field is busy finding solutions to these problems, the practitioner is faced with questions of how to integrate these findings into everyday work. A decision on whether to monitor collaboration, with which instrument and how often, is not easily made. It seems advisable to get informed about the client to continuously keep track of ongoing processes (Lambert et al., 2005; Kraus et al., 2011). In fact, it appears to be beneficial to monitor as closely and often as possible, because many therapeutically relevant processes change in stages, unpredictable and non-linear (Emmerling and Whelton, 2009; Halfon et al., 2016; Schiepek et al., 2016a).

The problem of bridging empirical findings on therapeutic relationship and collaboration into therapeutic practice becomes further aggravated in the field of families with a multitude of problems. Not only is family therapy in general faced with complex relationships between therapist, caregivers, and children (Shirk and Karver, 2003; Tuerk et al., 2012), multi-problem families (MPF) also show high drop-out rates, less compliance to tasks and goals agreed on to be part of therapy, such as filling in questionnaires. In addition, they are often faced with dramatic present and past life events which complicate any kind of intervention or maintaining good collaboration (Critchfield and Benjamin, 2006; Friedlander et al., 2006). Usually the goal-directed collaboration in the treatment of MPF in home-based treatments begins at “precontemplation or contemplation stage” with less “goal-directed collaboration” (subsequently we use the term collaboration). Too often families are not able or willing to collaborate in a goal-directed manner and therefore might be characterized as “unwilling, involuntarily or mandated” clients (Bachler et al., 2014). Friedlander et al. (2006) discriminate four types of clients in family psychotherapy (‘customers,’ ‘plaintiffs,’ ‘visitors,’ and ‘hostages’) with a great impact on goal directed collaboration. ‘Customers’ (motivated patients) are mainly to be found in outpatient psychotherapy. These patients show insight and a high willingness to work on their problems. ‘Plaintiffs,’ ‘visitors,’ and ‘hostages’ are the dominant types in home-based treatment for MPFs. These three groups of family dynamics share a lack of insight problem-perception and collaboration. ‘Plaintiffs’ have less insight to a problem but tend to complain without seeing that they are part of the problem. ‘Visitors’ believe that others are mistaken, and ‘hostages’ are resentful against the therapist for being confronted with problems by an allegedly hostile therapist. For MPF, goal directed collaboration and a good therapeutic relationship is therefore an important therapeutic factor, eventually forming the basis of therapeutic change. MPF are often characterized by, e.g., dysfunctional parental relationships, ego-structural and interactional family dynamic abnormalities with respect to the family’s everyday problem-solving abilities, bad emotional climate, and/or poor parenting skills of the primary caregiver. Studies on the epidemiology of MPF also show that this group of patients is generally hard to reach and therapy involves high costs (burden of disease) (Wittchen and Jacobi, 2005). Consequently, treatment concepts for MPFs are of great socio-political importance.

The aim of the present research is to help bridge the gap between empirical and practical considerations on how to monitor the therapeutic relationship in terms of collaboration and individual goals, especially for multi-problem families, and use monitoring data in therapy (Jacob et al., 2017b). To do so, our therapeutic/diagnostic approach contained an easy to use assessment of collaboration through the MPF-Collaboration Scale and attainment of individual goals by the therapist. Validating this method in line with the literature (Horvath et al., 2011; Tryon and Winograd, 2011), we hypothesized that (i) a positive change of collaboration scores and attainment of goals is connected to a better outcome and managing to stay in treatment without dropping out. Extending the approach of Hersoug et al. (2013), we tested this hypothesis by grouping clients into five groups of collaboration (deteriorating, stable low, stable medium, stable high and improving goal directed collaboration) and relating the groups to symptomatic change, individual goals, and treatment duration; a procedure that allows for a high face value and easy applicability to everyday practice.

Secondly, therapists should get informed as early as possible which clients are in special need of focussing on a good therapeutic relationship. Knowing who is most vulnerable in terms of dropping out of therapy due to stable low or deteriorating collaboration is of paramount interest to institutions, therapists, and clients themselves. We therefore tested exploratively, whether (ii) clients that fell into the groups of deteriorating or stable low collaboration as measured with the MPF-Collaboration Scale, had more severe family problems and a weaker expectancy of treatment at the beginning of treatment. Confirmation of that hypothesis also validates the MPF-Collaboration Scale, as it resembles earlier findings of, e.g., Constantino et al. (2011), who showed in their meta-analysis that patients’ expectations are of great importance for engaging in a goal-directed collaborative working relationship in different treatment settings with their therapists, which in turn improves treatment outcome. Furthermore, it has been demonstrated that patients who indicated more hopelessness show lower scores of outcome expectancy (Goldfarb, 2002). Vîslă et al. (2016) showed the bidirectional relation between outcome expectation and alliance. In addition, family adversities (FAI) are closely connected with functional impairment, severity of problems and interpersonal conflicts (measured by GAF, GARF) and suggest general pathways from family dysfunction to psychopathology (Raposo et al., 2013).

## Materials and Methods

### Treatment Procedure

The treatment method applied in this study – Therapeutic Outpatient Family Treatment (OFT) – was developed as a disorder-oriented, therapeutic outreach intervention for families with multiple problems. It integrates structural, family therapy interventions (Minuchin and Fishman, 1983), psychoanalytic elements of mentalization-based psychotherapy (Fonagy et al., 2006), and structural psychotherapy (Rudolf, 2006). OFT seeks to improve general parental skills of primary caregivers of minors through intra-psychological and interpersonal improvement of ego-structural skills, such as perception of self and others, defense and affect regulation, attachment, and communication (cf. Opd-2 Arbeitskreis Opd, 2006, Axis IV). The program incorporates the principles for treatment of personality disorders and structural psychotherapy (for the improvement of ego-structural competencies) that were identified by the task force of the APA Division 12: a strong working alliance, therapist ability to repair alliance ruptures, collaboration on goals, and a high level of therapist activity (Critchfield and Benjamin, 2006). Therapists at the institution have different therapeutic backgrounds (psychodynamic therapy and family therapy are in the majority) and obtain specific training in the integrative, technical characteristics of the OFT-approach. The therapeutic work takes place at the home of the families and in the natural environment of the index child. The costs of the treatment are borne by the Austrian or German Child Welfare Office, respectively. The average number of therapy hours in the institution and the sample constitutes 2.5–3 per week, divided amongst two sessions. Therapy is conducted by a group of 170 psychotherapists servicing 650 families.

### Measures

#### MPF (Multi-Problem Family)-Collaboration Scale

The MPF-collaboration scale is an integrated part of the routine assessment of OFT (Bachler et al., 2014). Therapists estimate and report on the collaboration, choosing one of five levels: (1) “The family has deep insight into its problems and shows continuously good goal directed collaboration.” (2) “The family recognizes itself as being part of the problems and is interested in understanding the problems, and shows mostly willingness to collaborate goal directed.” (3) “The family shows a passive recognition of own problems and a low to medium goal directed collaboration.” (4) “Problems are experienced as inflicted from the outside; involuntary goal directed collaboration; working together, but feeling forced to.” (5) “No insight, complete defense, neglect of problems, goals and goal directed collaboration; no willingness to collaborate.” The data are taken from a narrative interview the therapist conducts with the family, referencing to a defined list of three factors with various items building a total score. (1) Conduct and handling of tasks, such as acting out, coming too late, missing sessions, displaying boredom, aggressive transference, fatigue, and negative therapeutic response. (2) Content and form of communication, which can be inadequacy of affects, thematic fixation, avoidance of specific topics, rigidity, secrets, lack of examples, etc. (3) Therapeutic relationship, as e.g., different forms of transferences, depending on withdrawal, resistance, preliminary end of therapy caused by the patient, etc. The interrater reliability of MPF-collaboration has earlier been shown to be 0.75–0.87 and its correlation to the Heidelberg Structural Change Scale (HSCS) has been earlier found at 0.86, constituting a good criterium validity (Bachler, 2013). The interrater reliability of HSCS is 0.77–0.88 (Grande et al., 2009). In the present study, collaboration is assessed once every 6 months by the therapist involved as well as an external observer. This approach has been demonstrated to be more predictive for the outcome rates and more homogeneous in their ES than alliance measured by patients’ self-reporting (Bachelor, 2013).

#### Individual Therapeutic Goals (ITG)

The Individual Therapeutic Goal (ITG) rating follows the ITG module of the Psychotherapy Basis Documentation (PSYBADO; Heuft and Senf, 1998). It facilitates an individual definition of therapeutic goals that are important to the family as well as to the Child Welfare Office. The PSYBADO includes a standardized catalog of goals with five main categories: intrapsychic, interactional, somatic, addiction, and social medicine. The realization of the therapeutic goals is recorded graphically by the Goal Attainment Scaling (GAS) during treatment and is reflected in the supervision sessions. For the GAS, an inter-rater reliability of 0.82 has been reported (95% *CI* = 0.73–0.91; Steenbeek et al., 2010). Face-, construct-, and social-validity coefficients ranged from 0.62 to 0.83 (Turner-Stokes, 2011). In a study by Winter et al. (2005) the reliability (Cronbach’s α) of PSYBADO is estimated between 0.65 and 0.83. The construct validity there is reported with 0.82.

#### Treatment Outcome Expectations (VH-OFT)

VH is also an integrated part of the routine assessment of OFT. This five-point Likert scale rates one parameter of the family system with respect to the outcome expectancy, with high scores indicating low expectations (Bachler et al., 2014). The data of VH are taken from a narrative interview the therapist conducts with the family, referencing to a defined list of items. The interrater reliability of this rating scale is 0.79.

#### Family Adversity Index (FAI)

The Family Adversity Index (FAI; Rutter and Quinton, 1977) measures families’ psychosocial stress. Based on five items (chronic disharmony in the family, a low socioeconomic status, cramped living quarters, parental criminality, and mental disorder of the mother), the ensuing total value ranges from a minimum value of zero to a maximum value of five. Values ≥ 2 in the FAI reflect considerable socio-familial stress. Reliability has been found at 0.65, and validity in the range of 0.66 to 0.70 (Rutter and Quinton, 1977).

#### Mannheim Parental Interview (MPI)

The Mannheim Parental Interview (MPI; Esser et al., 1989) is a structured and standardized clinical interview indicating psychological disorders and their severity. The 37 questions regarding child and adolescent psychiatric symptoms combine a cumulative child-psychiatric symptom score and different ICD diagnoses. The interrater reliability is reported by the founders of the questionnaire between 0.71 and 1.0, the kappa coefficient (concurrence) of the diagnoses is 0.71 (percentage of concurrence 79% between professional judgements).

#### Global Assessment of Functioning Scale for Adults and Children (GAF, CGAF)

The Global Assessment of Functioning Scale (GAF), based on the DSM IV axis 5, is frequently employed in psychotherapy studies as a measure of disability and psychosocial dysfunction (Saß et al., 2003). Interrater reliability scores of 0.74 have been reported (Hilsenroth et al., 2000). The questionnaire is split into an adult version (GAF) and the CGAF for children (aged at 4 and above) and adolescents.

#### GARF Scale

The Global Assessment of Relational Functioning (GARF) assesses the psychosocial level of functioning of the families. It covers three dimensions: (i) problem solving; (ii) organization; and (iii) emotional climate (Stasch and Cierpka, 2006). The interrater reliability is 0.72, Cronbach’s alpha is 0.91, and the generalizability coefficient (GC) is 0.93. The validity coefficients range between 0.50 and 0.73, (Denton et al., 2010).

### Subjects (Inclusion/Exclusion Criteria of Families and Index-Child)

Therapeutic OFT generally started after a prior MPF classification by the Child Welfare Office. Data gathering started in 2008 and ended in 2015. The complete sample consisted of *N* = 810 adolescents. The average duration of therapy was 20.4 months (*SD* = 13.35, *Mdn* = 17.65, range = 1–74.5). The sample consisted of 422 boys (52.1%), 386 girls (index patients) and two cases where no contact to the index patient (child) could be established, who remained in the preliminary data-analysis as all other early drop-outs did. The mean age was 14.5 years (*SD* = 4.87, *Mdn* = 15.0, range = 1–24). A total of 169 therapists covered an average 4.8 cases (*SD* = 3.93; *Mdn* = 4; Min = 1; Max = 21).

At the beginning of the therapy 46% of the primary caregivers fulfilled the criteria for a personality disorder according to the MPI (Esser et al., 1989). The proportion of uneducated (without completed school education) primary caregivers in the clinical sample was 32%, so that a low wage ratio was to be expected; in particular, families with single-parent families belong to the group with the least social and personal resources (Franz et al., 2003). Personal and social resources are essential to develop child-psychiatric symptoms in the context of the risk increase for children and adolescents (Fryers and Brugha, 2013). The percentage of single parents was 47%. According to the FAI criteria the sample was characterized by the following features: Low socioeconomic status 33%, cramped living quarters 23%, chronic disharmony in the family 77%, parental criminality 7%, and severe mental disorder of the mother (primary caregiver) 58%. The average pre-treatment scores in the clinical sample were: GAF (primary caregiver) 6.1 (*SD* = 1.56), GARF 2.9 (*SD* = 1.89), CGAF 5.7 (*SD* = 1.1), and FAI 2.4 (*SD* = 1.1). MPF show an average GARF score of (*X* ≤ 4.7); “families without risk” an average score of (*X* = 6.4) (Stasch and Cierpka, 2006).

From the complete sample, 368 families finished the treatment as planned and by mutual acceptance of clients and therapists. 127 families stopped the treatment due to placement of the child in institutions or foster families. Failure to comply with previously agreed terms of treatment was the reason for 150 families to drop out earlier than originally planned (e.g., due to a high number of failed therapy-sessions), 36 families moved outside our sphere of influence and 29 families are combined under a group that terminated treatment for a mixture of “other reasons.”

## Results

### Intercorrelations of the Measures

**Table 1** shows the bivariate correlation matrix of the pre-treatment measures and the respective reliability coefficients of the rating scales, which are all in the acceptable range for Cronbach’s alpha and interrater reliability, except for FAI, which is moderately reliable (Cronbach, 1951; McHugh, 2012). There are significant positive correlations of the treatment alliance with treatment expectations [*r*(810) = 0.613, *p* < 0.001] and the FAI [*r*(810) = 0.260, *p* < 0.001]. A negative correlation was found between the baseline scores of MPF Collaboration Scale and the individual therapeutic goals ITG [*r*(753) = -0.154, *p* < 0.001]. Differences in number of participants per scale are due to differences in assessment guidelines of the respective instruments (e.g., MPI is only assessable above an age of 4). A detailed description of the pre- and post-scores and a comparison of these in terms of *t*-tests can be found in Bachler et al. (2014).

**Table 1:** Bivariate correlation matrix of pre-treatment measures and reliability coefficients (on the main diagonal).

Pre-treatment measures	1	2	3	4	5	6	7	8
(1) MPF Collaboration Scale	(0.75)”							
(2) Treatment expectation (VH-TAF)	0.613^∗∗^ *N* = 810	(0.79)”						
(3) Individual therapeutic goals	-0.154^∗∗^ *N* = 753	-0.232^∗∗^ *N* = 753	(0.76)’					
**Psycho-social health:**								
(4) Family adversity index (FAI)	0.260^∗∗^ *N* = 810	0.287^∗∗^ *N* = 810	-0.105^∗∗^ *N* = 753	(0.65)’				
(5) Severity of problems (MPI)	0.058 *N* = 792	0.081^∗^ *N* = 792	-0.05 *N* = 747	0.014 *N* = 792	(0.71)’			
(6) Caregiver (GAF)	0.008 *N* = 747	0.019 *N* = 747	0.003 *N* = 704	-0.043 *N* = 747	-0.015 *N* = 735	(0.74)’		
(7) Child (CGAF)	0.088^∗^ *N* = 532	-0.002 *N* = 532	-0.079 *N* = 495	0.031 *N* = 532	-0.072 *N* = 523	0.402^∗∗^ *N* = 532	(0.74)’	
(8) Relation (GARF)	0.047 *N* = 572	0.026 *N* = 529	-0.017 *N* = 529	-0.064 *N* = 572	-0.03 *N* = 572	0.520^∗∗^ *N* = 572	0.451^∗∗^ *N* = 532	(0.82)’


*^∗^Significant at 0.05; ^∗∗^significant at 0.01; ’Cronbach’s alpha reliability coefficient; ”inter-rater reliability*.

The focus of this paper is on patterns of change of collaboration scores and the respective parameters. By subtracting the pre- from the post-scores, difference scores were produced for each parameter. The difference score of the collaboration scale significantly correlates – as hypothesized – with the change scores of all other parameters, except for the CGAF (**Table 2**); the bivariate Pearson’s correlations of the change scores for collaboration show a significant reduction of barriers towards treatment expectation, *r*(810) = -0.638, *p* < 0.001, decrease of the FAI, *r*(810) = -0.316, *p* < 0.001 and less severity of problems, *r*(792) = -0.169, *p* < 0.001. Furthermore, patients who positively changed in terms of their collaboration scores also reported to progress more towards their ITG, *r*(753) = 0.442, *p* < 0.001, improve the global assessment of the caregiver *r*(747) = 0.096, *p* < 0.001 and advance in terms of the family’s relational functioning *r*(572) = 0.134, *p* < 0.001.

**Table 2:** Bivariate correlation matrix of change scores.

	1	2	3	4	5	6	7
(1) MPF Collaboration Scale							
(2) Treatment expectation (VH-TAF)	-0.638ˆ**						
(3) Individual therapeutic goals	0.442ˆ**	-0.538					
**Psycho-social health:**							
(4) Family adversity index (FAI)	-0.316ˆ**	0.446ˆ**	-0.401ˆ**				
(5) Severity of problems (MPI)	-0.169ˆ**	0.195ˆ**	-0.264ˆ**	0.126ˆ**			
(6) Caregiver (GAF)	0.096ˆ**	-0.034	0.046	0.007	-0.043		
(7) Child (CGAF)	0.077	-0.059	0.045	0.027	-0.094ˆ*	0.461^∗∗^	
(8) Relation (GARF)	0.134ˆ**	-0.082ˆ*	0.08	-0.016	-0.041	0.522^∗∗^	0.544^∗∗^


*^∗^Significant at 0.05; ^∗∗^significant at 0.01*.

### Process of Group Production

The discrimination of collaboration patterns is based on the difference scores of the collaboration ratings (Hersoug et al., 2013). Computing the Reliable Change Index (RCI) with 0.68 for the collaboration variable, a decrease/increase of 1.33 points was used to identify five different collaboration groups: Patients in the deteriorating collaboration (*N* = 48; 5.9% of the complete sample) reduced their collaboration by -4 to -2 and patients in the improving collaboration group (*N* = 170; 21.0%) had an increase of collaboration of at least +2 (**Table 3**). Patients whose collaboration did not change more the ±1.33 points were clustered into the three stable collaboration groups, according to their level of collaboration: stable low collaboration (*N* = 228; 28.1%; collaboration level > 3), stable medium collaboration (*N* = 202; 24.9%; collaboration level = 3), stable high collaboration (*N* = 162; 20.0%; collaboration level < 3) (Jacobson et al., 1984; Wise, 2004). These groups were used for the subsequent data analysis.

**Table 3:** Number of patients in the five clusters of alliance per collaboration pre-post difference scores.

	Pre-post difference scores of collaboration										
Collaboration group	-4	-3	-2	-1	0	1	2	3	4	Total
Decreasing collaboration	2	7	39	0	0	0	0	0	0	40
Stable low collaboration	0	0	0	38	89	101	0	0	0	228
Stable medium collaboration	0	0	0	27	85	90	0	0	0	202
Stable high collaboration	0	0	0	28	84	50	0	0	0	162
Increasing collaboration	0	0	0	0	0	0	123	40	7	170
**Total**	**2**	**7**	**39**	**93**	**258**	**241**	**123**	**40**	**7**	**810**

*Stable low collaboration: collaboration level > 3; stable medium collaboration: collaboration level = 3; stable high collaboration: collaboration level < 3*.

### ANOVA and *Post hoc* Contrasts

**FIGURE 1:**
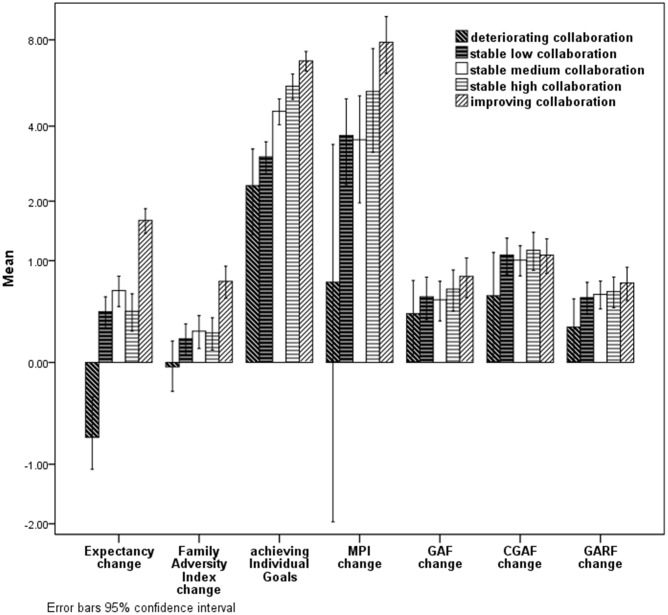
Change scores of the respective outcome measures per collaboration group.

**Figure 1** shows the mean change scores of all outcome variables, split for the respective collaboration groups. Conducting a one-way independent ANOVA, there was a significant effect of belonging to a specific collaboration group on the treatment effects for the MPI [*F*(4,787) = 5.1, *p* < 0.001] and for achieving individual goals [*F*(4,748) = 52.8, *p* < 0.001]. The Levene’s test of homogeneity of variances revealed differences for the change of FAI [*Levene statistic* (4,805) = 18.94, *p* < 0.001] and Treatment Expectancy [*Levene statistic* (4,805) = 4.01, *p* = 0.003]. After using the Brown-Forsythe test to counter the inequality of variance due to the difference in group sizes by adjusting the degrees of freedom, the respective collaboration group still differed significantly for the FAI scores [*F*(4,648.6) = 21.9, *p* < 0.001] and also on the expectancy change [*F*(4,406.7) = 72.3, *p* < 0.001].

Planned contrasts revealed that the groups of stable low collaboration and decreasing collaboration as compared to the groups of stable high collaboration and increasing collaboration (with stable medium collaboration set to 0 in these contrasts) reached significantly less change on the MPI [*t*(787) = 4.0, *p* < 0.001, *d* = 0.31], on achieving individual goals [*t*(748) = -12.6, *p* < 0.001, *d* = 1.17], on the FAI [with variances assumed unequal; *t*(335.6) = 7.0, *p* < 0.001, *d* = 0.55] and the treatment expectancy [*t*(118.8) = 12.3, *p* < 0.001, *d* = 0.96]. Families with increasing collaboration or good collaboration thus attained significantly more progress in terms of achieving their goals (ITG), their treatment expectancy (VH-OFT), reducing family problems (FAI) and severity of child psychiatric symptoms (MPI).

### Treatment Length and Dropout per Group

The mean length of the treatments was 20.4 months (*SD* = 13.3). When comparing the collaboration groups, differences between the groups in terms of treatment length were found [*F*(4,804) = 20.4, *p* < 0.001]. With a mean of 27.2 month (*SD* = 14), patients who fell into the improving collaboration group had a significantly longer treatment duration compared to all other groups, as Tukey HSD *post hoc* tests revealed. In particular, the treatment length of the improving collaboration group differed from the deteriorating collaboration group (*M* = 16.2 months, *SD* = 10.2, *p* < 0.001, *d* = 0.83), the stable low collaboration group (*M* = 16.5 months, *SD* = 12.2, *p*< 0.001, *d* = 0.83), the stable medium collaboration group (*M* = 18.7 months, *SD* = 11.5, *p* < 0.001, *d* = 0.67), and the stable high collaboration group (*M* = 22.1 months, *SD* = 14.1, *p* < 0.001, *d* = 0.37). In short, the better the collaboration, the longer the treatment duration.

When comparing the different collaboration groups in terms of the respective reason to end treatment, ‘failed compliance’ accounted for the majority of 66% of the patients in the deteriorating collaboration group. In contrast, the majority of cases in the improving and stable high collaboration groups ended treatment ‘as planned,’ with 58.6 and 71.4% per respective group.

### Predictors

To test for possible predictors of the respective collaboration groups, we conducted a multinomial logistic regression for the criterion of belonging to one of three groups: high collaboration (combined of stable high collaboration and improving collaboration), medium collaboration, and low collaboration (combined of stable low collaboration and deteriorating collaboration). When entering all initial scores of the measures into the model, we find the initial scores of treatment expectancy (*b* = 0.54, Wald χ^2^(1) = 18.5, *p* < 0.001) and the initial scores of the FAI (*b* = 0.43, Wald χ^2^ (1) = 14.8, *p* < 0.001) to significantly predict whether a patient falls into the high collaboration group or in the low collaboration group. The odds ratio produced the information that per 1-point increase of the initial values of treatment expectancy and FAI, the odds of being in the low collaboration group increased by 1.72 [*CI*: 1.34–2.2] and 1.5 [*CI*: 1.23–1.9], respectively. Thus, the lower the expectancy (that is a high VH value) and the higher the adversity scores in a family, the more likely these were to fall in the low collaboration cluster.

## Discussion

Collaboration as one of the core ingredients of psychotherapy differs across patients and within patients in the course of a therapy. In our study, a group of 810 patients could be categorized into 5 change clusters of collaboration: Stable high collaboration (occurs with 20%), improving collaboration (2.1%), stable low collaboration (28.1%), deteriorating collaboration (5.9%), and stable medium collaboration (24.9%). These clusters not only differed in their evolution of collaboration, but also showed significant differences in terms of achieving ITG (*d* = 1.17; *r* = 0.50), changing expectancy of treatment (VH-OFT, *d* = 0.96; *r* = 0.43), and decreasing problems within the family (FAI, *d* = 0.55; *r* = 0.26). A change of the severity of child-psychiatric symptoms was established (MPI, *d* = 0.31; *r* = 0.15), even though the sample includes high rates of diagnoses like adolescent personality disorders and Asperger-syndrome with lower therapeutic variability (e.g., ICD 10 F60 21%, sever forms of ADHD 19%, and ICD 10 F84-89 special diagnoses 7% of the sample). Taken together, patients who manage to maintain a good collaboration or improve collaboration show a higher impact on therapeutic change in terms of outcome and for the achievement of individually defined treatment goals than those who do not.

Our data show that clients who fell into the groups of families with deteriorating and stable low collaboration were more likely to have low pre-treatment expectations and more familial adversities (interpersonal functioning, pre-treatment symptom level, social/economic status; FAI and VH-OFT). Such alarming pre-treatment scores might therefore be interpreted as an early signal to therapists to focus on the improvement of the collaboration.

Hopelessness in clients has earlier been shown to be related to decreased post-treatment outcomes in psychotherapy (Constantino et al., 2011; overall weighted effect size of *d* = 0.24; *r* = 0.11). Similarly, outcome expectancy (VH-OFT) was correlated in the present sample with collaboration (*r* = 0.64; *d* = 1.60), as well as with individual goal attainment (ITG; *d* = 1.2; *r* = 0.54). One might argue that outcome expectation and less general adversity in a family’s surrounding (FAI) fuel the willingness and ability for cooperation (collaboration), which results in reaching individually set goals (ITG). Similarly, Sharabi et al. (2012) showed the clinically relevant connection between family climate, hopelessness, and child development risks in low SES families.

The accumulation and severity of problems, combined with a lack of hope and often cross-generational failures to improve the family’s situation, are obstacles for MPFs to start and maintain therapy. Bischoff and Sprenkle (1993) showed connections between drop-out rates and low SES, which has been shown to be predictive for premature termination of treatment (Petry et al., 2000). In our sample, patients with improving collaboration reached the longest treatment duration as well as the highest chance to end treatment as planned. Meta-analysis by Swift and Greenberg (2012) shows a termination rate of 19.7% for adults in psychotherapy, with the specific rate depending on the diagnoses of the patients (Axis II), their ages, and the experience of the therapists (number of studies examined: 699; patient random sample *N* = 83,834). In child and adolescent psychotherapy, there is a higher drop-out rate than in adult therapy, ranging from 28 to 85% (Garcia and Weisz, 2002). Our sample shows a total drop-out rate of 18.5%. This low drop-out rate is likely due to the setting of homebased treatment.

### Limitations

The study follows a naturalistic design and the treatments were conducted within the usual practice of our institute. In consequence, causal interpretations are, in a strict sense, not possible. But our findings are of high external validity and thus generalizable, especially for long-term home-based treatments with MPF. Descriptive validity (study documentation, use of a case record form, treatment description, and data protection) is given, and treatment adherence was checked. There are only minor threats on the construct validity (to some extent separation of data collection and treatment). Possible rater-biases constitute a limitation for all ratings based on observers.

## Conclusion

Our data add a strong relationship between collaboration and the achievement of ITG of *d* = 0.98; *r* = 0.44 (weighted effect size for “goal directed collaboration” of *d* = 0.69; *r* = 0.33; Tryon and Winograd, 2011). Friedlander et al. (2011) showed similar results for family-therapy (*d* = 0.49; *r* = 0.24). The present study emphasizes the impact of the collaboration and shows its importance in therapeutic work with MPF, especially in relation to *treatment outcome expectancy* and *adversities within a family*. Changing expectancy of treatment (VH-OFT, *d* = 0.96; *r* = 0.43), and decreasing problems within the family (FAI, *d* = 0.55; *r* = 0.26), are important influencing family-(patient)-related factors in the treatment of MPF, explaining 38,9% (collaboration 19,3%, expectancy of outcome 12,9%, decreasing problems within the family 6,7%) of the variance of individual goal achievement (ITG). Their influence in the treatment of MPF is higher than in other therapeutic settings for various other patient groups.

### Research Results and Clinical Implications

With respect to the therapeutic implications, our results suggest that fostering and improving the therapeutic relationship through collaboration is of high importance to the therapeutic process of home-based treatment with MPF (hard to reach families). This statement is supported by the result that the change of collaboration explains 40.7% of change in treatment expectancy and 10% of the change in family adversity. Therefore, a fine-tuning of each individual change process according to the present state of the therapeutic relationship and adapting interventions accordingly might help to increase the willingness of patients and their ability to collaborate. Therapists’ activities in fostering the collaboration on goals is influenced bidirectional by a number of further relevant evidence-based technical adaptive features in the treatment of therapy in treatment of MPF: Handling RRPs (rupture repair processes) and thereby fostering the development of the therapeutic bond, emphasizing change by adaptive therapist activity (flexibility, availability and treatment intensity), setting rules and boundaries (consensus on task), processing maladaptive intrapsychic and interpersonal thoughts, behavior and parenting (Lebow, 2005; Bachler et al., 2014); and, as our data show, improving outcome expectations (hope) by encouraging patients and by supporting them in solving adversities within the family. In particular, differentiating “stable collaboration” (low, medium high stable) from improving and deteriorating collaboration helps explaining the therapeutic outcome and offers a valid approach to everyday practice. It stresses the importance of treatment-monitoring (Schiepek et al., 2016b).

The present study is a contribution to treatment aptitude research (Norcross and Wampold, 2011). It intends to widen the empirical evidence and to bridge the gap towards an applicable approach for the practice. Reacting to differences amongst the group of families with multiple problems, it appears favorable to individually tailor treatments using empirical results and pre-treatment characteristics of patients; a notion in line with the Presidential Task Force on Evidence-Based Practice (American Psychological Association, 2006, p. 273), which emphasized the importance of research on patient characteristics, culture, social classes, and preferences for the future of psychotherapy.

## Ethics Statement

This study was carried out in accordance with the recommendations of WMA Declaration of Helsinki – Ethical Principles for Medical Research Involving Human Subjects. All subjects gave written informed consent in accordance with the Declaration of Helsinki. The study received ethical approval from the Government of Salzburg.

## Author Contributions

All authors added Contributions to Study design, Proofread and Literatur. BA was responsible for Statistics. EB and AF for data Collection. EB, BA, and GS for writing the paper.

## Conflict of Interest Statement

The authors declare that the research was conducted in the absence of any commercial or financial relationships that could be construed as a potential conflict of interest.
